# Molecular, morphological, and life history data to support research of huntsman spiders (Araneae: Sparassidae)

**DOI:** 10.1016/j.dib.2023.108885

**Published:** 2023-01-06

**Authors:** Jacob A. Gorneau, Linda S. Rayor, Cristina A. Rheims, Corrie S. Moreau

**Affiliations:** a Department of Entomology, Cornell University, Ithaca, NY, United States 14853; b Institute for Biodiversity Science and Sustainability, California Academy of Sciences, 55 Music Concourse Drive, San Francisco, CA, United States 94118; c Laboratório de Coleções Zoológicas, Instituto Butantan, Av. Vital Brasil, 1500, 05503-900 São Paulo, SP, Brazil; d Department of Ecology and Evolutionary Biology, Cornell University, Ithaca, NY, United States 14853

**Keywords:** Molecular phylogenetics, Deleninae, Social evolution, Behavior, Life history, Spider biology

## Abstract

This article on biodiversity and life history data in huntsman spiders (Araneae: Sparassidae) includes the following: molecular data deposited on GenBank for 72 individuals representing 27 species in seven subfamilies, life history and behavioral data on 40 huntsman species from over two decades of observations, and morphological data for 26 species in the subfamily Deleninae as well as an undescribed representative of the genus *Damastes*. Molecular data include the nuclear genes histone H3 (H3) and 28S ribosomal RNA (28S rRNA), mitochondrial genes cytochrome c oxidase subunit I (COI) and 16S ribosomal RNA (16S rRNA) were sequenced via Sanger sequencing by J.A. Gorneau. Life history data were collected in the field and in the lab by L.S. Rayor and include data on age at sexual maturity, lifespan, social classification, egg sac shape, how the egg sac is attached or carried, retreat location, retreat modification, retreat size relative to adult female body size, approximate mean body mass, and mean cephalothorax width. Morphological data on Deleninae and one *Damastes* sp. were scored by C.A. Rheims and includes information on the following characters: prosoma (fovea, posterior eye row shape (PER), anterior median eye (AME) diameter, AME-AME and PME-PME interdistances), male palp (embolic sclerite (PS), conductor sclerotized base (SB), tegular apophysis (TA), flange (f)) and female epigyne and vulva (epigynal sclerite (ES), spermathecal sacs (SS)). These data were used to clarify relationships among the Australian endemic Deleninae, as well as global patterns in sparassid evolution. The data demonstrate phylogenetic patterns in life history, social evolution, and natural history among the sparassids. These data contribute to future comparative research on sparassid systematics, evolution, and behavior. This data article complements a research article published in Molecular Phylogenetics and Evolution [1].

**Specifications Table**

**Table:** 

Subject	Systematics, Ecology, and Behavior
Specific subject area	Arachnology: Evolution, Morphology, and Life History
Type of data	Multiple gene sequences, morphological character matrix, life history character matrix
How the data were acquired	Molecular data: Sanger sequencing completed at the Cornell Genomics Facility of the Biotechnology Resource Center (BRC; Ithaca, NY, USA). ABI sequence files were assembled de novo using the program Geneious Prime 2020.0.5. Morphological data: Specimens were examined immersed in 70% alcohol, under a LEICA MZ 12.5 stereomicroscope (Leica Microsystems, Germany). Illustrations were made with the aid of a camera lucida. Behavioral and life history data: Field and lab observations of wild-caught huntsman spiders collected by LSR in Australia (2002 – 2021) and Singapore (2017) using appropriate state and national permits. Specimens were collected in Australian Capital Territory (ACT), New South Wales (NSW), Victoria (VIC), Tasmania (TAS), Western Australia (WA), and (Queensland (QLD). Additionally, live specimens imported through the pet trade from Africa, Madagascar, and Asia were included in the study. In the lab, mother-offspring groups were housed in round plastic or glass aquarium enclosures to observe early interactions and the behavior of developing offspring as described in Yip et al. [2].
Data format	Assembled gene sequences for four loci (H3, 28S rRNA, COI, 16S rRNA), MAFFT-aligned sequence files in NEXUS (.nex) and PHYLIP (.phy) format as required by each inference program, morphological characters in tabular format, life history characters in tabular format.
Description of data collection	DNA extracted from 27 species of huntsman spiders for use in Sanger sequencing; morphological data for 26 species of Deleninae and *Damastes* scored by C.A. Rheims, life history data for 40 species (primarily Heteropodinae and Deleninae) collected and scored by L.S. Rayor.
Data source location	Voucher representatives for molecular work (which also includes species examined for behavioral and life history traits) stored at the Smithsonian National Museum of Natural History, Washington, District of Columbia, United States of America (NMNH) and information regarding these specimens is viewable in the Zenodo dataset (**Voucher_information.xlsx**).
	Voucher representatives for morphological work stored at the Instituto Butantan, São Paulo, Brazil (IBSP); Queensland Museum, Brisbane, Australia (QSM); Western Australian Museum, Perth, Australia (WAM) and information regarding these specimens is viewable in the Zenodo dataset (**Morphology_data.xlsx**).
Data accessibility	Aligned sequence data in .nex and .phy format, morphological and life history data in comma-separated values (.csv) format, and input files, log files, and code for all analyses are included on Zenodo and described here. Sequences are available from GenBank using the accession numbers provided in Table 1. Repository Name: Zenodo Direct URL to data: https://doi.org/10.5281/zenodo.7249925.
Related research article	J.A. Gorneau, C.A. Rheims, C.S. Moreau, L.S. Rayor, 2022. Huntsman spider phylogeny informs evolution of life history, egg sacs, and morphology. Mol. Phylogenet. Evol. 174, 107530. https://doi.org/10.1016/j.ympev.2022.107530.

## Value of the Data

• While focused molecular investigations and life history (including natural history and behaviour) datasets are not uncommon, providing an integrated dataset with both molecular and life history data allows for examination of trends in the context of evolution.
• These data will be of particular use to arachnologists, evolutionary biologists, systematists, behavioral ecologists, and biogeographers looking to explore trends in comparative social evolution and life history, morphology, and more generally, the evolution of the Sparassidae and Australian endemism.
• Molecular data deposited in GenBank will be available for future studies of molecular evolution and phylogenetic analyses, as well as for species-based identification using the barcode gene cytochrome c oxidase subunit I (COI).
• Voucher exemplar specimens for molecular, morphological, and behavioral data are deposited in museums for replicability and additional analysis, including sequencing of additional loci in the future.
• Morphological and behavioral data will inform individuals designing their own character matrices for members of the Sparassidae, as well as provide a basis from which to designate and define certain life history characteristics.
• Long-term datasets presenting total evidence character traits are a rich source for researchers to design their own character matrices for comparative study.

## 1 Data Description

**Fig. 1:**
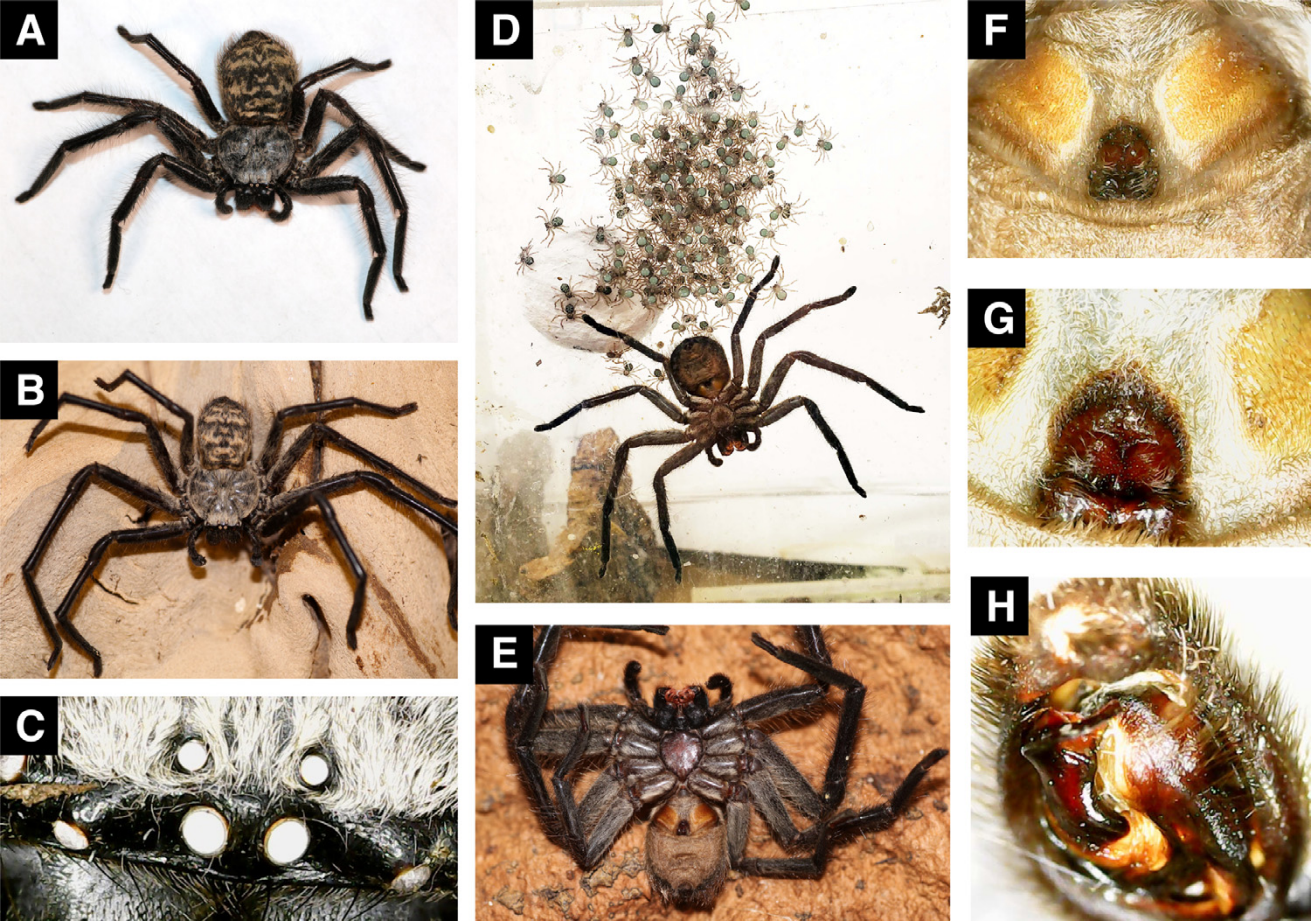
Images of the unidentified prolonged subsocial *Damastes* species used this study. A: adult female. B: adult male. C: recurved eye row of adult male*.* D: adult female with first instar young and plastered egg sac. E: adult female venter with epigynum. F, G: images of the epigynum. H: adult male right palp.

These data present a detailed compilation of molecular, morphological, life history, and behavioral character states for representatives of 37 of the 89 genera of Sparassidae, the eleventh-most speciose spider family, and focus on taxa endemic to Australia. We provide tables including accession numbers for sequences contributed to GenBank, morphological and life history character matrices, and input files and code for each analysis. We also include R code in R Markdown (.Rmd) format for the phylogenetic comparative methods used in Gorneau et al. [1], input files for IQ-TREE, RAxML, MrBayes, and BEAST phylogenetic inferences. See captions for more information on Fig. 1 and Table 1.

**Table 1:** Table with GenBank accession numbers for new data generated in Gorneau et al. [1]. Red cells indicate no sequence data for that gene, and gray cells indicate sequence data but only from a single sequencing direction (either forward or reverse). More information can be found in **New_data_generated_GenBank.xlsx** in the Zenodo dataset.

## 2 Material Available on Zenodo

### 2.1 Protocol, Voucher, and GenBank Files

**Primers_and_PCR_protocols.xlsx** — Excel file with information on primers used in this study. C1-N-2776 was used for samples for which the HCO/LCO combination of primers did not amplify adequate DNA. Second sheet of file includes information on PCR protocols used.
**Voucher_information.xlsx** — Excel file with information on vouchers representing exemplars for the molecular data (which also includes species examined for behavioral and life history traits) contributed in this study. Specimens deposited in the National Museum of Natural History (NMNH, USA) arachnology collections.
**New_data_generated_GenBank.xlsx —** Excel file that corresponds to Table 1 with more detailed information on individuals for which new sequences were generated.
**GenBank_sequences.xlsx** — Excel file with accession numbers for sequences downloaded from GenBank.

### 2.2 IQ-TREE Files

**IQTREE_Sparassidae_10_Nov_2021.phy** — Input file for IQ-TREE inference containing concatenated alignment for all four gene sequences of taxa used in this analysis.
**IQTREE_partition.nex** — Input NEXUS file for IQ-TREE inference containing details on partitioning of four genes in concatenated dataset **IQTREE_Sparassidae_10_Nov_2021.phy**.
**IQTREE_10_Nov_2021.log** — Output .log file for IQ-TREE inference containing details of run progress and models of molecular evolution selected.
**IQTREE_10_Nov_2021.contree** — Output IQ-TREE phylogeny with results of 10,000 ultrafast bootstrap replicates as nodal support values.

### 2.3 RAxML Files

**RAxML_Sparassidae_10_November.phy.raxml.startTree —** Starting tree as inferred by IQ-TREE. The same tree file as IQTREE_10_Nov_2021.contree.
**RAxML_22_Nov_2021_partition.txt —** inference containing details on partitioning of four genes in concatenated dataset.
**RAxML_Sparassidae_10_Nov_2021.phy —** Input file for RAxML inference containing concatenated alignment for all four gene sequences of taxa used in this analysis.
**RAxML_Sparassidae_10_Nov_2021.raxml.log —** Output .log file for RAxML inference containing details of run progress and models of molecular evolution selected.
**RAxML_Sparassidae_10_Nov_2021.raxml.bestTree —** Best maximum likelihood tree as inferred by RAxML with bootstrap values as nodal support values.

### 2.4 MrBayes Files

**MrBayes_21_Dec_2021.nex** — Input NEXUS file for MrBayes inference with concatenated dataset of four genes, MrBayes block with information about sequence partitions and models used, as well as IQ-TREE phylogeny used as starting tree.
**MrBayes_21_Dec_2021_stout.txt** — Output file with information on MrBayes run including average standard deviation of split frequencies for duration of run.
**MrBayes_21_Dec_2021.tre** — Bayesian inference phylogeny output of MrBayes with posterior probability values as nodal support values.

### 2.5 Tree Convergence Files

**Tree_convergence_huntsman.Rmd** — R Markdown file with code to analyze convergence between IQ-TREE inferences and MrBayes and RAxML inferences. Employs the use of the phytools R package.

### 2.6 BEAST Files

**BEAST_28_Jan_2022_mono.xml** — Input file for BEAST 2.6.0 containing information about priors for divergence dating including models of molecular evolution and fossil outgroup calibrations. This file was run independently three times through BEAST to generate three independent .log and files that were then combined into **BEAST_28_Jan_2022_combined_runs_123.log** in the program LogCombiner.
**BEAST_28_Jan_2022_combined_runs_123.log** — The combined .log files for the three independent runs of BEAST. Contains information about effective sample sizes to determine the quality of the run in BEAST 2.6.0. Examined using Tracer v.1.7.1
**BEAST_28_Jan_combined_123_TA_25.tre** — Maximum clade credibility tree from three independent BEAST runs identified by TreeAnnotator, with input merged .trees files from three runs and burn-in percentage 25%. Node heights set at keep target heights.

### 2.7 Life History, Stochastic Character Mapping, and D-test Files

**Life_history_data.xlsx** — Excel file containing two spreadsheets: one named Character_States with information about character states for nine life history characters, and one named Character_Matrix with character matrix of life history attributes.
**lh_matrix.csv** — .csv file with same character matrix from **Life_history_data.xlsx** Excel file, for use in stochastic character mapping and D-test code in **Stochastic_character_mapping_and_D-test_huntsman.Rmd**.
**lifeHistorysubName.csv** — .csv file with substitutions of names from IQ-TREE phylogeny tip labels to names for stochastic character mapping; essentially removes specific identifiers for ease of visualization in **Stochastic_character_mapping_and_D-test_huntsman.Rmd** code.
**Stochastic_character_mapping_and_D-test_huntsman.Rmd** — R Markdown file containing code for stochastic character mapping of life history and D-test correlation analysis. Requires the use of the following R packages: corHMM, phylotools, and phytools. Also available on GitHub.
**Stochastic_character_mapping_model_selection.csv** — .csv file with output of model selection including Akaike information criterion (AIC) values from **Stochastic_character_mapping_and_D-test_huntsman.Rmd** code among the following models: equal rates (ER), symmetric (SYM), and all rates different (ARD) for all life history characters.
**D_test_p_values.xlsx** — Excel file with output partitioned by individual sheet showing the p-values of the D-test analysis in a pairwise matrix. The numeric codes for character states correspond to the following file: **Life_history_data.xlsx**.
**Stochastic_character_mapping_posterior_probabilities.xlsx** — Posterior probabilities of stochastic character mapping analyses organized by character and then by individual node. Node numbers correspond to **Stochastic_character_mapping_w_node_numbers**.
**Stochastic_character_mapping_w_node_numbers.pdf** — PDF file with results of stochastic character mapping analyses with node numbers so **Stochastic_character_mapping_posterior_probabilities.**xlsx can be consulted to examine specific posterior probabilities for specific life history characters by node.

### 2.8 Morphology Files

**Morphology_data.xlsx** — Excel file with four sheets: Character_States describing the characters and character states in the Character_Matrix sheet; the Character_Matrix sheet, which is the basis for the input file **morpho_matrix.csv**; Morphological_Vouchers sheet with information on specimens directly examined for morphological character scoring; and Morpohology_From_Literature for species scored with the use of literature, either instead of direct examination or in tandem with direct observation.
**morpho_matrix.csv** — .csv file of character matrix from **Morphology_data.xlsx** slightly altered such that the questionable characters were marked as the characters they are likely to be for ease of plotting using the code in **Morphology_huntsman.Rmd**. The uncertainty was then readded in Adobe Illustrator.
**morphosubName.csv** — .csv file with substitutions of names from IQ-TREE phylogeny tip labels to names for mapping of morphological data matrix from **morpho_matrix.csv**; essentially removes the specific identifiers for ease of visualization in **Stochastic_character_mapping_and_D-test_huntsman.Rmd** code.
**Morphology_huntsman.Rmd** — R Markdown file containing code for mapping data matrix of morphological data Deleninae + *Damastes*. Input files: **morpho_matrix.csv, morphosubName.csv, IQTREE_10_Nov_2021.contree**. Employs the use of the following packages: corHMM, phylotools, phytools, and RColorBrewer. Also available on GitHub.
**Male_genitalia.tif** – Male, left palp, ventral view. A: *Beregama cordata* (L. Koch, 1875). B: *Delena cancerides* Walckenaer, 1837. C: *Holconia flindersi* Hirst, 1991. D: *Isopeda villosa* L. Koch, 1875. E: *Isopedella leai* (Hogg, 1903). F: *Neosparassus salacius* (L. Koch, 1875). G: *Pediana regina* (L. Koch, 1875). H: *Typostola barbata* (L. Koch, 1875). I: *Zachria flavicoma* L. Koch, 1875. Scale lines: 1 mm. F = tegular flange; PS = palpal embolic sclerite; SB = conductor sclerotized base; TA = Deleninae tegular apophysis
**Female_genitalia.tif** – Female, genitalia. A–B: *Delena cancerides* Walckenaer, 1837 (A: epigyne, B: vulva). C–D: *Holconia flindersi* Hirst, 1991 (C: epigyne, D: vulva). E–F: *Isopeda villosa* L. Koch, 1875 (E: epigyne, F: vulva). G–H: *Isopedella conspersa* (L. Koch, 1875) (G epigyne, H vulva). I–J: *Isopedella leai* (Hogg, 1903) (I: epigyne, J: vulva). K–L: *Pediana regina* (L. Koch, 1875) (K: epigyne, L vulva). M–N: *Typostola barbata* (L. Koch, 1875) (M: Epigyne, N: vulva). O–P: *Zachria flavicoma* L. Koch, 1875 (O: epigyne, P: vulva). Scale lines: 1 mm. ES = epigynal sclerite; SS = spermathecal sac.

## 3 Experimental Design, Materials and Methods

### 3.1 Specimen Observation, Care, and Collection

Behavior and life history traits for each species were observed in the field and/or laboratory. Spiders were fed a diet of crickets (*Gryllodes sigillatus* and *Acheta domesticus*), houseflies (*Musca domestica*), calliphorid flies (*Calliphora* spp.), and fruit flies (*Drosophila* spp.). A table with specimen information and voucher information for exemplars of molecular work is included in the Zenodo dataset (**Voucher_information.xlsx**). Legs (or whole bodies, where spiderlings were used) from individuals were stored in ethanol and placed in a -20°C freezer prior to DNA extraction.

### 3.2 DNA Extraction, Amplification, and Sequencing

A total of 54 samples were extracted in fall 2019 by JAG using the QIAGEN DNeasy PowerSoil Kit (Qiagen, Inc., Valencia, CA, USA). A single adult leg or whole bodies of immatures were used for DNA extraction. An additional 30 samples were extracted by Dr. Ingi Agnarsson's lab group at the University of Vermont using the QIAGEN DNeasy Tissue Kit (Qiagen, Inc., Valencia, CA, USA). DNA from these extractions was then amplified using polymerase chain reaction (PCR) on a BioRad 96-well C1000 Touch Thermal Cycler (BioRad, Hercules, CA, USA) for two mitochondrial genes (cytochrome c oxidase subunit I (COI) and 16S ribosomal RNA (16S rRNA) and two nuclear genes (histone H3 (H3) and 28S ribosomal RNA (28S rRNA). In short, PCR reactions involved 6.5 mL water, 12.5 mL EconoTaq® PLUS Master Mix (Lucigen, Middleton, WI), 2.5 mL each of forward and reverse primer (10 mM), and 1 mL of DNA for each 25 mL reaction. The primers used for each gene in this study, and PCR conditions for each gene can be found in the Zenodo dataset (**Primers_and_PCR_protocols.xlsx**). PCR products were visualized using gel electrophoresis on a 1% agarose gel with 2 ml of DNA and 2 ml GelRed dye under a BioRad UV transilluminator and imaged using the ImageLab™ software (BioRad, Hercules, CA, USA). PCR products were purified using ExoSAP-IT (Thermo Fisher, Waltham, MA, USA) to remove remaining primers and dNTPs. Purified PCR samples were quantified using a Qubit 4 fluorometer (Thermo Fisher, Waltham, MA, USA) and Biotium High Sensitivity AccuGreen dye (Thermo Fisher, Waltham, MA, USA) to determine the quantity of DNA after purification and immediately prior to sequencing. Samples were cycle-sequenced in the forward and reverse directions and diluted based on the DNA concentration for full-service (post-PCR purification) sequencing at the Cornell Genomics Facility (Ithaca, NY, USA).

### 3.3 Sequence Data from Previous Studies

In addition, sequences for a total of 201 samples were downloaded from GenBank (Zenodo, **GenBank_sequences.xlsx**).

### 3.4 Sequence Assembly and Alignment

Contigs were made from the forward and reverse sequencing products where possible, and where not possible, only forward or reverse sequences were used (single-stranded sequences represented 28 sequences, approximately 11% of all sequences, Table 1). These sequences were then exported to FASTA format for use in downstream analyses. Accession number information is available in Table 1. Sequences were combined in a single file with those obtained from GenBank, and multiple sequence alignments for each gene was performed using MAFFT on the CIPRES Science Gateway [3]. For the H3 and COI genes the L-INS-i method was used, while for the variable ribosomal genes the E-INS-i method was used as recommended by Wheeler et al. [4]. Multiple sequence alignments were examined manually, and for protein coding genes, alignments were further assessed using the minimize stop codons visual in Mesquite. Once this was complete, anomalous or repetitive regions deemed not phylogenetically informative due to these regions lacking sequence data in at least 90% of other sequences were removed as they lacked phylogenetic signal.

### 3.5 Partitioning and Phylogenetic Inferences

Aligned sequences were partitioned and exported from Mesquite in .phy format for analysis in IQ-TREE version 1.6.12 [5]. The following models were selected in IQ-TREE using ModelFinder: GTR+F+I+G for COI, K2P+I+G4 for H3, TIM2e+R4 for 28S rRNA, TIM2+F+I+G4 for 16S rRNA [5,6]. An IQ-TREE maximum likelihood inference was performed using an ultrafast bootstrap approach (UFBoot) with 10,000 bootstraps, with 25% of the trees discarded as burn-in [5,7]. The output tree was rooted with outgroups *Deinopis spinosa* Marx 1889 (Deinopidae), *Uloborus diversus* Marx 1898 (in Banks 1898; (Uloboridae)), *Oecobius* Blackwall 1862 (Oecobiidae), *Uroctea durandi* ((Latreille 1809); Oecobiidae), *Peucetia viridans* ((Hentz 1832); Oxyopidae), *Dolomedes tenebrosus* Hentz 1844 (Pisauridae), *Salticus scenicus* (Clerck 1757; Salticidae), *Selenops muehlmannorum* Jäger & Praxaysombath 2011 (Selenopidae), and *Tibellus chamberlini* Gertsch 1933 (Thomisidae). The IQ-TREE inference was used as a starting tree for inferences in RAxML and MrBayes with the concatenated sequences partititioned by gene to see if the tree topologies were comparable [8,9]. The code in the Zenodo dataset (Tree_convergence_huntsman.Rmd) visually examines convergences among RAxML and MrBayes runs with IQ-TREE.

### 3.6 BEAST Estimation of Divergence Time

An estimation of divergence time was conducted in BEAST using fossil calibrations recommended for use by Magalhães et al. [10,11]. The fossils used in this calibration were *Zamilia aculeopectens* Wunderlich 2015 (Oecobiidae) for the node representing Oecobiidae, *Oxyopes succini* Petrunkevitch 1958 (Oxyopidae) for Oxyopidae + Pisauridae, *Almolinus ligula* Wunderlich 2004 (Salticidae) for the node containing the outgroups Salticidae + Thomisidae, and ‘*Selenops*’ sp. indet. Wunderlich 1988 (Selenopidae) for the node with outgroups Selenopidae sister to Salticidae + Thomisidae [10]. For *Zamilia aculeopectens*, an exponential distribution was set with an offset of 98.17 and a mean of 0.32. For *Oxyopes succini* and *Almolinus ligula*, an exponential distribution was set with an offset of 43.0 my, and a mean of 1.3. For ‘*Selenops*’ sp. indet., an exponential distribution was set with an offset of 53.0 my, and a mean of 0.8. As such, these analyses were conducted in accordance with the latest work on fossil calibration of phylogenies by Magalhães et al. [10]. The partitions were set by running individual genes in jModelTest2 and using the models best suited for Bayesian information criterion were implemented in BEAST, with the GTR+I+G model used for all genes [12]. The selected clock model was relaxed clock log normal with -1 discrete rates and an estimated clock rate of 5.3E-4. For the tree model, a birth death model was used. All outgroup priors were constrained as monophyletic. The IQ-TREE phylogeny was used as a starting tree. The preceding settings were input to BEAUti for 100,000,000 generations and an XML input file was created for use in the program BEAST version 2.6.0 [11] on the CIPRES Science Gateway. The XML file was run through BEAST three times to increase effective sample size (ESS) and ensure the results represented a global rather than local optimum. The results of the BEAST analyses were evaluated in Tracer, where ESS values were examined. The tree samples were summarized and 25% burn-in was set in TreeAnnotator.

### 3.7 Life History and Habitat Data

From 2002 – 2021, LSR collected life history and behavioral data from 40 sparassid species, with emphasis on the endemic Australian Deleninae. Data was collected in the field (Australia, Singapore) and in her laboratory at Cornell University (USA). Life history variables included: mother-offspring dynamics and sociality, egg sac structure, how the egg sac was attached to the retreat or carried, retreat type, modifications to the retreat, adult female body mass and cephalothorax width, age at sexual maturity, and lifespan. These character states are outlined in the Zenodo dataset (**Life_history_data.xlsx)**.

Social classification was based on the duration of association and complexity of social interactions between mothers and their offspring prior to sexual maturity. ‘Solitary’ species dispersed within three weeks (late first instar or the second instar post-emergence from the egg sac), ‘subsocial’ species dispersed between four – five weeks (third or fourth instar), ‘prolonged subsocial’ species remained in mother-offspring groups for five to twelve months (fifth to ninth instar, or after sexual maturity in the tenth instar) prior to dispersing.

Three types of egg sacs were observed in the species studied: ‘plastered’ with a ground sheet silked onto the substrate and the rest of the sac built onto that attached sheet, a ‘lenticular’ (a relatively flat round disc), and a ‘spherical’ (ball-like) shape. Egg sacs varied in support structure among the sparassids studied: egg sacs were either ‘completely adhered’ to the substrate and immobile, ‘tethered’ by guy-lines of silk and relatively immobile, or actively ‘carried’ by the adult female under her venter. Adhered egg sacs were only accessible on one side, while tethered and carried egg sacs were relatively accessible on both sides. The spiders in this analysis used retreats under tree ‘bark’ or in small hollows in trees, ‘rocks’, in ‘dead foliage’, in ‘living foliage’, or in the open without a retreat (‘in the open’). Modifications included either ‘silk bonds’ whch are repeated short silken swaths that bind the bark/rock/leaves together, effectively limiting access to the retreat, or a small ‘silken cage’ that completely surrounds the female and her egg sac forming a retreat, or ‘none’ in which there was no silk modification of the retreat.

Additionally, parameters of body size (mean body mass, cephalothorax width), age of female at sexual maturity, and average life span in captivity were collected.

### 3.8 Stochastic Character Mapping of Life history Traits and D-test for Correlation

To investigate the evolution of life history in the context of phylogeny, the inferred IQ-TREE phylogeny tips were trimmed using the keep.tip function in the R package phytools to the 40 species for which life history data as collected by LSR existed [13]. To avoid any biases due to branch lengths, the tree was converted to ultrametric. Prior to conducting stochastic character mapping, model selection was conducted for each life history trait, through the three basic models (equal rates, all rates different, and symmetric): social classification, how egg sac attached or carried, egg sac structure, retreat location, retreat modification, retreat size relative to body size, retreat size with eggs, approximate mass, and approximate cephalothorax width. Stochastic character mapping was implemented for the estimation of ancestral states at each node of the phylogeny for 40 sparassid species with data on the following life history traits. Stochastic maps were generated using a Markov-chain Monte Carlo (MCMC) approach for 1000 generations, with sampling every ten generations, for a total of 100 stochastic map trees generated. From these stochastic map trees posterior probabilities for each node were generated. The D-test was implemented for 100 generations using the phytools function Dtest to examine correlations between sociality and each of the following life history traits: how egg sac attached or carried, egg sac structure, retreat location, retreat modification, retreat size relative to body size, retreat size with eggs, approximate mass, and approximate cephalothorax width [13,14]. This test has previously been used to examine correlations between morphological data but is used here to examine for correlations between sociality in life history [13,14].

### 3.9 Morphological Data for Deleninae and *Damastes*

Relevant morphological data for the endemic Australian Deleninae and *Damastes* were tabulated in a matrix. Character scoring was based on direct examination of available specimens (Zenodo dataset, **Morphology_data.xlsx**), and when unavailable, from literature [15], [16], [17], [18], [19], [20]. The file also contains collection information on specimens for character scoring. Examined material belongs to the following institutions (abbreviation and curator in parentheses): Instituto Butantan, São Paulo, Brazil (IBSP, A.D. Brescovit), Queensland Museum, Brisbane, Australia (QMS, R. Raven); Western Australian Museum, Perth, Australia (WAM, M. Harvey). Left male palps were detached from the body and illustrated in ventral view (Zenodo dataset, **Male_genitalia.tif**). Female epigynes were dissected and illustrated in ventral and dorsal views. In dorsal view illustrations, the hyaline part of the copulatory ducts was omitted (Zenodo dataset, **Female_genitalia.tif**).

## Ethics Statement

This work is consistent with the ethical requirements and standards for publication. This study did not include any human subjects, data collected from social media platforms, or animal experiments requiring approval. The authors have no conflict of interest to disclose.

## CRediT authorship contribution statement

**Jacob A. Gorneau:** Conceptualization, Data curation, Formal analysis, Funding acquisition, Investigation, Methodology, Project administration, Software, Validation, Visualization, Writing – original draft, Writing – review & editing. **Linda S. Rayor:** Conceptualization, Data curation, Formal analysis, Funding acquisition, Investigation, Methodology, Project administration, Resources, Supervision, Validation, Writing – original draft, Writing – review & editing. **Cristina A. Rheims:** Data curation, Formal analysis, Funding acquisition, Investigation, Methodology, Resources, Supervision, Validation, Writing – review & editing. **Corrie S. Moreau:** Conceptualization, Formal analysis, Funding acquisition, Investigation, Methodology, Project administration, Resources, Supervision, Writing – review & editing.

## Declaration of Competing Interest

The authors declare that they have no known competing financial interests or personal relationships that could have appeared to influence the work reported in this paper.

## Data Availability

Molecular, morphological, and life history data to support research of huntsman spiders (Araneae: Sparassidae) (Original data) (Zenodo).
